# Transgenerational plasticity alters parasite fitness in changing environments

**DOI:** 10.1017/S0031182022001056

**Published:** 2022-09

**Authors:** Syuan-Jyun Sun, Marcin K. Dziuba, Kristina M. Mclntire, Riley N. Jaye, Meghan A. Duffy

**Affiliations:** 1 Department of Ecology & Evolutionary Biology, University of Michigan, Ann Arbor, MI 48109, USA; 2 International Degree Program in Climate Change and Sustainable Development, National Taiwan University, Taipei 10617, Taiwan

**Keywords:** Climate change, *Daphnia dentifera*, host–parasite interactions, *Metschnikowia bicuspidate*, priming, transgenerational plasticity

## Abstract

Transgenerational plasticity can help organisms respond rapidly to changing environments. Most prior studies of transgenerational plasticity in host–parasite interactions have focused on the host, leaving us with a limited understanding of transgenerational plasticity of parasites. We tested whether exposure to elevated temperatures while spores are developing can modify the ability of those spores to infect new hosts, as well as the growth and virulence of the next generation of parasites in the new host. We exposed *Daphnia dentifera* to its naturally co-occurring fungal parasite *Metschnikowia bicuspidata*, rearing the parasite at cooler (20°C) or warmer (24°C) temperatures and then, factorially, using those spores to infect at 20 and 24°C. Infections by parasites reared at warmer past temperatures produced more mature spores, but only when the current infections were at cooler temperatures. Moreover, the percentage of mature spores was impacted by both rearing and current temperatures, and was highest for infections with spores reared in a warmer environment that infected hosts in a cooler environment. In contrast, virulence was influenced only by current temperatures. These results demonstrate transgenerational plasticity of parasites in response to temperature changes, with fitness impacts that are dependent on both past and current environments.

## Graphical abstract

**Figure:**
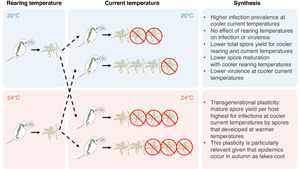

## Introduction

In the face of rapid environmental changes, avoiding extinction requires organisms to respond with behavioural changes, moving to a new geographic region, rapid evolution and/or phenotypic plasticity (Hoffmann and Sgrò, 2011; Munday *et al*., 2013; Wong and Candolin, 2015; Radchuk *et al*., 2019). For organisms with limited behaviours and mobility, phenotypic plasticity has the potential to be particularly important, as it allows for a rapid response without genetic changes. Thus, for many organisms, phenotypic plasticity is expected to play a key role in allowing organisms to cope with rapidly changing environments (Charmantier *et al*., 2008; Wong and Candolin, 2015).

Moreover, phenotypic plasticity can facilitate rapid evolutionary processes, including by buying time for genetic adaptations to occur (Mousseau and Fox, 1998; Sun *et al*., 2020). This can be particularly true when a plastic response to environmental changes can be passed from one generation to the next, something known as transgenerational plasticity or maternal effects (Harmon and Pfennig, 2021). Transgenerational plasticity has been demonstrated in many taxa; often, previous generations ‘prime’ their offspring for environmental conditions that match those of maternal generations (Mousseau and Fox, 1998; Burgess and Marshall, 2014). If the parental environments are accurate predictors for the environments of offspring, transgenerational plasticity can be adaptive, increasing the fitness of offspring experiencing those environmental conditions (Galloway and Etterson, 2007).

Although transgenerational plasticity is increasingly considered as a potentially important mechanism to counteract the negative impacts of environmental changes, most studies have focused on responses of a single species to changing abiotic environments, such as temperature elevation (Shama *et al*., 2014) or environmental pollution (Tran *et al*., 2019; Meng *et al*., 2021). However, changing environments also have the potential to influence the way organisms interact. Focusing on just one type of interaction, host–parasite interactions, increasing temperatures can alter parasite reproduction, infectivity and prevalence, host resistance and ultimately epidemic dynamics (Harvell *et al*., 2002; Altizer *et al*., 2013; Gehman *et al*., 2018). While this likely leads to selection on parasites in many cases, transgenerational plasticity may also play a role in allowing parasites to persist in a rapidly changing environment. There have been some studies of transgenerational plasticity in host–parasite interactions, but most of the attention has been focused on hosts (Gervasi *et al*., 2015; Pigeault *et al*., 2015; Michel *et al*., 2016; Nystrand *et al*., 2016; Roth and Landis, 2017; Paraskevopoulou *et al*., 2022) rather than on parasites or the overall outcome of host–parasite interactions (Tseng, 2006; Little *et al*., 2007; Shocket *et al*., 2018). This means that, currently, there is a major gap in our understanding of how host–parasite interactions and parasite fitness change as environments change, hindering our ability to understand and predict epidemic dynamics in a rapidly changing world.

In addition to its importance in light of anthropogenic climate change, understanding transgenerational plasticity of parasites is important because seasonal changes in environments are common and can influence the outcomes of host–parasite interactions within a generation (Altizer *et al*., 2006; Martinez, 2018). Changing temperatures can have complex impacts on hosts and parasites (Altizer *et al*., 2013), making it difficult to predict *a priori* how parasitism should change seasonally (Altizer *et al*., 2006). One factor that increases the complexity even further is that hosts might encounter parasites that were produced in previous seasons (or even years) under different environmental conditions (Decaestecker *et al*., 2004; Shocket *et al*., 2018). Overall, it is clear that changes in temperature can strongly impact host–parasite interactions; in some systems, hosts encounter parasites that were produced at different temperatures; and the conditions under which a parasite develops can impact its infectivity. However, at present, it is not clear whether parasite transgenerational plasticity influences key parasite traits such as virulence.

In this study, we tested whether the temperature at which parasite spores develop influences performance of their offspring using a 2-generational laboratory experiment in a zooplankton–fungal system. *Metschnikowia bicuspidata* is a fungal parasite commonly found infecting the zooplankton host *Daphnia dentifera* in freshwater lakes in Northern America (Cáceres *et al*., 2014). In this system, *D. dentifera* is exposed to *M. bicuspidata* spores during filter feeding. The ingested needle-shaped spores then penetrate through the gut barrier into the body cavity (Stewart Merrill *et al*., 2019), where the spores develop and reproduce, eventually killing the host (Ebert, 2005). Upon host death, the spores of the next generation are released into the environment, where they can be ingested by new hosts to complete the infection cycle (Ebert, 2005). Previous work shows that *D. dentifera* and *M. bicuspidata* are likely to encounter each other during autumnal epidemics: infection prevalence tends to increase when temperatures cool from 25°C in the late summer, with infection prevalence peaking at 20°C, and decreasing as temperatures drop during winter (Shocket *et al*., 2018). Moreover, it is likely that spores overwinter in the sediment, with some of these becoming resuspended and infecting new hosts in future years (Ebert, 1995; Decaestecker *et al*., 2002); this means that spores produced at the end of one season (in colder conditions) might infect a host at warmer temperatures in a subsequent year. Thus, we would expect that *D. dentifera* can be exposed to *M. bicuspidata* spores derived from warmer or cooler thermal conditions. If transgenerational plasticity exists, we predict that parasites reared at different temperatures should perform differently; if such transgenerational effects are adaptive, we predict that offspring will perform better when their temperature matches that of their parent. In contrast, if transgenerational plasticity is not important, only the current temperatures, rather than rearing temperatures, should influence parasite performance. Prior studies have shown that rearing environments (namely, host genotype and temperature) can influence the infectivity of *M. bicuspidata* spores (Searle *et al*., 2015; Shocket *et al*., 2018); this shows plasticity of parasites but not transgenerational plasticity, given that the spores from the rearing conditions are the same ones that infect the new hosts. Here, we tested for transgenerational plasticity impacting parasite growth and virulence.

## Materials and methods

### Experimental design

We conducted a fully factorial experiment by exposing *D. dentifera* hosts to *M. bicuspidata* spores: the spores were reared either at cooler (20°C) or warmer temperature (24°C) and then used factorially for exposures at 20 or 24°C. The 4°C elevation of temperatures that we studied is in line with the predicted climate change scenario by the end of this century (Beits *et al*., 2011). It is also well within the range of changes in temperature in the upper mixed layer of lakes that occur during epidemics (Shocket *et al*., 2018). In total, this resulted in 4 treatment combinations of rearing and current temperatures, with 40 replicates per treatment. Laboratory stocks of *D. dentifera* and *M. bicuspidata* originated from lakes in Barry County in Michigan, USA.

As infections will occur at the ambient temperature, and because hosts will have been reared at that temperature, we reared hosts for this experiment at the 2 focal temperatures. More specifically, we reared *D. dentifera* at 20 or 24°C in only 2 separate incubators (I-41VL, Percival Scientific, Perry, IA, USA) for 2 generations on a 16 : 8 photoperiod; this means that temperature treatment is confounded with the incubator (as is common in this type of experiment due to logistic constraints). We then collected neonates aged 1–2 days old. Each neonate was maintained individually in a 50 mL beaker filled with 50 mL filtered lake water and was fed thrice a week with phytoplankton food (*Ankistrodesmus falcatus*, 20 000 cells mL^−1^).

To create sources of *M. bicuspidata* spores from different temperatures, we infected *D. dentifera* individually by adding spores at a density of 145 spores mL^−1^ at 20 and 24°C. We collected the dead hosts upon natural death, stored them individually in a 1.5 mL tube filled with 100 *μ*L deionized water, and placed the tubes in a refrigerator before use (*M. bicuspidata* spores die when placed in a freezer; Duffy and Hunsberger, 2019). All spores used for the experiment were derived from hosts that had died 1–2 months prior to use in infections; these infected hosts were the product of an earlier experiment (Sun *et al*., 2022). Infected hosts were ground individually using electric pestles for 60 s before we infected new hosts with a well-mixed solution of spores (145 spores mL^−1^) from each tube at 20 and 24°C. We used a degree-day approach (Vale *et al*., 2008; Manzi *et al*., 2020) by adding spores to *Daphnia* at ages of 6 and 5 days, for 20 and 24°C, respectively, resulting in a 120 degree-day. At exposure, we fed all animals with 20 000 cells mL^−1^ *A. falcatus* and maintained them at a 16 : 8 light : dark cycle. Thereafter, all animals were fed with *A. falcatus* (20 000 cells mL^−1^) thrice a week. We terminated the experiments when all animals had died. This experiment was conducted in March 2022.

### Data collection

To determine virulence, we checked all animals daily for survival and counted the offspring produced, which were then removed from the beakers. We determined the lifetime fecundity of hosts (the total number of offspring) and host lifespan; both lifespan and fecundity tend to be reduced by *M. bicuspidata* infections (Clay *et al*., 2019). The dead animals were stored individually in a 1.5 mL tube filled with 100 *μ*L deionized water, and were placed in a refrigerator for subsequent measurement of spore yield, a key component of parasite fitness. Because hosts contain all of the parasite spores that were produced over the course of infection at host death, we quantified spore yield, i.e. the number of spores per host, by grinding dead infected hosts with an electric pellet pestle (Fisher Scientific, Hampton, NH, USA catalogue no.: 12-141-361) for 60 s. A sample (10 *μ*L) of this solution was added to a Neubauer haemocytometer, and we estimated the spore yield by averaging the number of mature spores and total spores (mature + immature spores) from 4 grids. Mature spores can be visually distinguished from immature ones (Ebert, 2005; Stewart Merrill *et al*., 2019); mature ascospores were characterized by their needle shape, each containing a dark band in the centre, whereas conidia and needle-shaped spores lacking the central bands and sharp edge were considered immature (see Fig. S1 for examples).

*Daphnia* that died within 7 days after exposure to parasites were excluded because of early mortality. Any male *Daphnia* that were misidentified as females in the beginning of the experiment were also excluded. This resulted in a total of 141 individuals (current temperature of 20°C: *n* = 37 and 37 for rearing temperature of 20 and 24°C, respectively; current temperature of 24°C: *n* = 35 and 32 for rearing temperature of 20 and 24°C, respectively).

### Statistical analyses

To test for the effects of rearing and current temperature on host fitness, we included rearing temperature (20 or 24°C) and current temperature (20 or 24°C) and their interaction as fixed factors. We analysed the lifetime fecundity using a generalized linear mixed model (GLMM) with a Poisson distribution, and analysed the survival with a Cox proportional hazard mixed effect model. In both analyses, the parental source of the spores (i.e. the identity of infected host individual) was included as a random effect. We included this random effect because spores were derived from different host individuals; spores from a single individual were used to infect pairs of new hosts (one at 20°C and one at 24°C), with spores from a single individual used to infect 1–3 pairs of hosts.

We analysed the effects of rearing and current temperatures on parasite fitness in a similar fashion by including rearing and current temperatures and their interaction as fixed factors. We analysed the number of mature spores and total number of spores using GLMMs with a Gaussian distribution; we calculated the natural log of the number of spores plus 1 prior to analyses to meet the requirements of normality for regressions. We analysed the probability of infection and the proportion of spore maturation (the number of mature spores divided by the total number of spores) using GLMMs with a binomial distribution and logit link function. Similarly, the parental source of the spores was included as a random effect.

GLMMs were conducted with the glmer function in the *lme4* package (Bates *et al*., 2015), whereas the Cox proportional hazard mixed effect model was conducted in the *coxme* package (Therneau, 2012). We started the analyses by including the interaction terms, with non-significant interactions removed from the models. If significant interaction terms were detected, pairwise post-hoc comparisons were made to assess differences between individual treatments in the *emmeans* package (Lenth, 2021). All analyses were conducted in R version 4.1.2 (R Development Core Team, 2014).

## Results

**Fig. 1.**
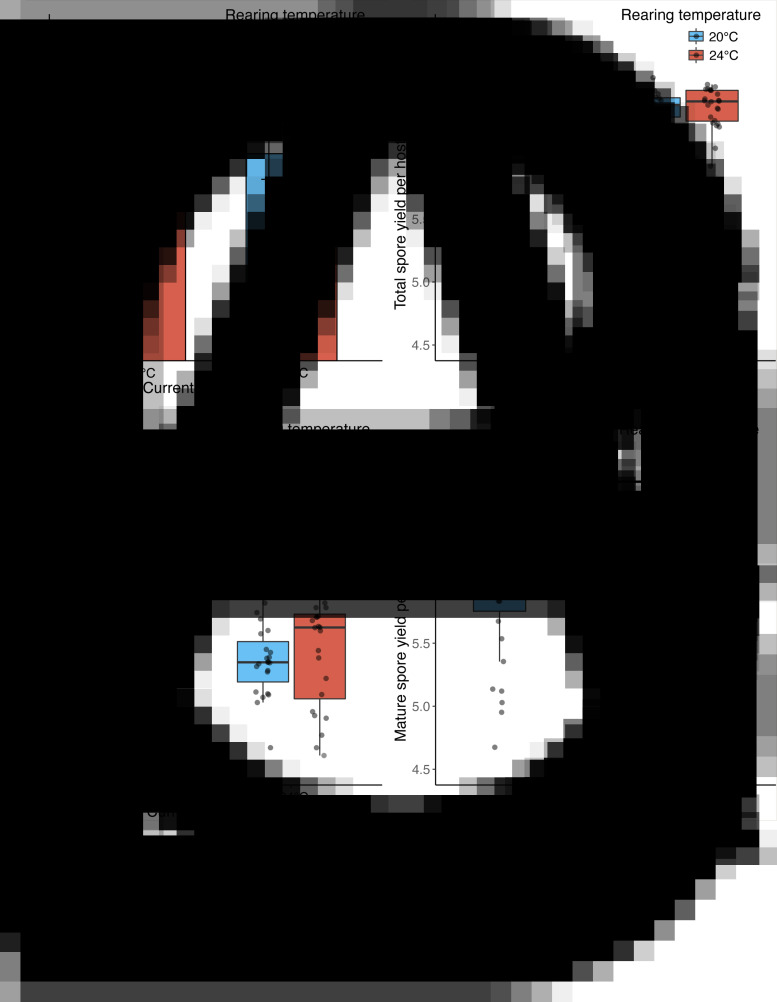
Parasite fitness was affected by both rearing and current temperatures, indicating transgenerational effects of temperature on parasites. Infection prevalence (A) was not as strongly impacted by temperature as was parasite fitness, measured as the number of total spore yield (B), proportion of spore maturation (C) and mature spore yield (D). Means and standard error bars are shown. The box plots show median values, the 25th and 75th percentiles and interquartile ranges. Significant (*) and non-significant (ns) differences between treatments are indicated in panel (D).

There was no effect of rearing temperature but a marginally significant effect of current temperature on infection prevalence: infections carried out at warmer temperatures led to a marginally lower infection prevalence than cooler temperatures (*χ*^2^ = 3.29, *P* = 0.070), regardless of whether the spores were reared at cooler or warmer temperature (Fig. 1A; *χ*^2^ = 0.13, *P* = 0.721). Among 141 *D. dentifera* exposed to *M. bicuspidata* spores, 82.4% (61 out of 74) and 70.1% (47 out of 67) developed terminal infection at current cooler and warmer temperatures, respectively.

For hosts that were successfully infected, both current (*χ*^2^ = 9.40, *P* = 0.002) and rearing (*χ*^2^ = 5.58, *P* = 0.018) warmer temperatures increased total spore yield (Fig. 1B). Of the spores produced, a smaller proportion showed maturity when current temperatures were warmer (59.2% at 20°C *vs* 36.3% at 24°C; *χ*^2^ = 33.22, *P* < 0.001) and when parasites were reared at cooler temperatures (46.5% at 20°C *vs* 51.9% at 24°C; *χ*^2^ = 6.74, *P* = 0.009; Fig. 1C). Moreover, current and rearing temperatures interacted in affecting the mature spore yield per host – i.e. the yield of spores that had reached the developmental stage with the potential to initiate a new infection (current temperatures × rearing temperatures: *χ*^2^ = 4.62, *P* = 0.032; Fig. 1D). Specifically, when infecting hosts at cooler temperatures, parasites that developed at warmer temperatures produced more mature spores compared to those that developed at cooler temperatures (*t* = −3.30, *P* = 0.002), yet there was no such difference between spores that developed at cooler *vs* warmer temperatures when infecting hosts at warmer temperatures (*t* = −0.14, *P* = 0.891; Fig. 1D). For parasites that developed at cooler temperatures, spore yield was similar at both cooler and warmer current temperatures (*t* = −0.30, *P* = 0.765; compare blue bars in Fig. 1D). However, parasites reared at warmer temperatures produced fewer mature spores at current warmer temperatures compared to current cooler temperatures (*t* = 2.73, *P* = 0.008; compare red bars in Fig. 1D).

**Fig. 2.**
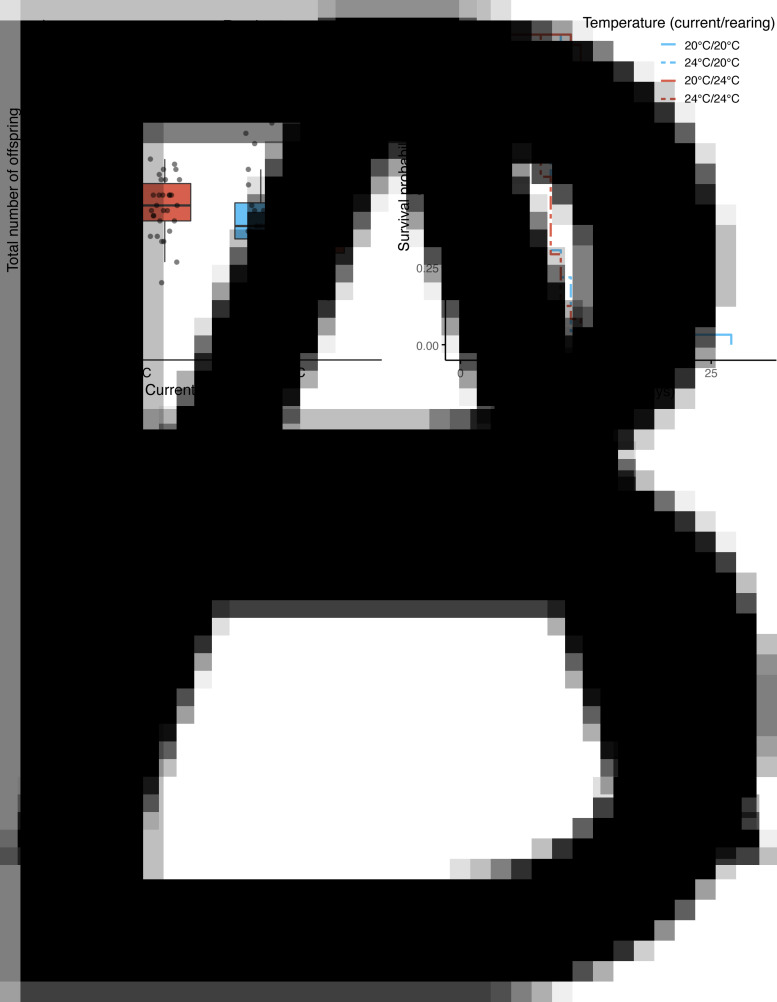
Virulence was affected by current temperature but not rearing temperature. This held both for virulence measured as the total number of offspring (A) and as survival probability (B). The colours indicate temperatures at which parasites were reared. The box plot shows median values, the 25th and 75th percentiles and interquartile ranges.

In contrast to the results for spore yield and maturation, parasite virulence was influenced by current temperature but not by rearing temperature (Fig. 2). There was no effect of the temperature at which parasites were reared on the total number of host offspring (*χ*^2^ = 1.45, d.f. = 1, *P* *=* 0.228). Fecundity was only affected by current temperatures, with fewer host offspring produced at warmer temperatures (*χ*^2^ = 10.67, d.f. = 1, *P* *=* 0.001; Fig. 2A). Similar patterns were observed in survival probability, such that there was no effect of rearing temperatures (*χ*^2^ = 1.13, d.f. = 1, *P* *=* 0.288) but a significant effect of current temperatures – infected individuals were more likely to die early at warmer temperatures (*χ*^2^ = 76.36, d.f. = 1, *P* < 0.001; Fig. 2B).

## Discussion

Transgenerational plasticity has the potential to influence organisms' responses to rapidly changing environments, yet whether transgenerational plasticity of parasites exists, and whether it alters parasite fitness and/or virulence, have been overlooked. By investigating the impacts of temperature on both rearing and current environments, we showed that infection prevalence depended mainly on current temperatures, whereas reproductive success of the parasite was determined by the interplay between the (rearing) temperature at which spores developed and the (current) temperature at which the new infections occurred. Infections by parasites that had been reared at warmer temperatures yielded more mature spores, but only at cooler current temperatures. Thus, our findings demonstrate transgenerational plasticity of parasites in response to changing temperatures but does not provide evidence for adaptive ‘priming’ of parasites.

We found that the prevalence of infection was explained by offspring infection temperatures but not by rearing temperatures; this suggests that *M. bicuspidata* spores were equally viable, irrespective of previous temperatures. This finding is consistent with previous findings (Little *et al*., 2007) on *Daphnia magna* and their interactions with the bacterial parasite *Pasteuria ramosa*, one of the best-studied parasites of *Daphnia*. A prior study found that current temperatures, rather than parasite rearing temperatures, influenced parasite infectivity, with highest infections at 20°C (Vale *et al*., 2008). Moreover, spore production per host was also highest at this temperature (Vale *et al*., 2008). In another study, optimal performance of the microsporidium parasite *Ordospora colligata* showed similar patterns (Kunze *et al*., 2022), peaking at ~20°C and decreasing at both higher and lower ranges of temperature gradient. Our results are also in line with previous work on the *Daphnia*–*Metschnikowia* system in that higher current temperatures reduced parasite infectivity (Shocket *et al*., 2018). However, we did not find the same pattern in the effect of rearing temperatures. The earlier study found that higher rearing temperatures increased infectivity (Shocket *et al*., 2018), whereas we did not find a significant effect of rearing temperature on infectivity; one possible explanation for this difference is that the earlier study explored rearing temperatures of 15–22°C whereas in our experiment temperature spanned from 20 to 24°C to simulate a shift towards a higher temperature range. Future work considering a wider range of temperatures would help to uncover whether spores reared at intermediate temperatures are most infectious.

Contrary to a previous study which showed that temperature warming can reduce spore production (Vale *et al*., 2008), we found that parasites actually had greater total spore yield (inclusive of both mature and immature spores) in response to warmer current temperatures. Parasites also produced more spores when the spores that began the infection had been reared at warmer temperatures. That is, warming induced parasites to be more productive. However, higher current and rearing temperatures had opposite effects on spore maturation (Fig. 1C): warmer current temperatures substantially reduced spore maturation, but warmer rearing temperatures increased spore maturation. The additive temperature effects of rearing and current temperatures on total spore yield per host, and their contrasting effects on the proportion of spores that matured, mean that warmer rearing temperatures enabled parasites to produce more mature spores than those reared at cooler temperatures, but only in cooler current environments. Yet we found no evidence to suggest that this transgenerational plasticity was adaptive, since the offspring of parasites reared at high temperatures did not have a fitness advantage at current high temperatures (Fig. 1D). These results corroborate previous studies showing the vulnerability of parasites to sustained exposure over generations to high temperatures (Carlson *et al*., 2017; Schampera *et al*., 2022). These findings might explain why seasonal epidemics of *M. bicuspidata* tend to erupt when lake temperatures gradually cool down in late summer (Shocket *et al*., 2018); in these cooling conditions, the transgenerational plasticity we observed would increase parasite fitness.

A potential caveat arises from the fact that the environment a parasite experiences very early in infection can influence parasite fitness (Röder *et al*., 2008), and this developmental plasticity could be hard to distinguish from transgenerational plasticity, particularly if the parental conditions influence the likelihood of infection. For example, if more spores successfully infected a host, that might have an impact on the final spore yield, although whether it would lead to more spores (because of a larger initial population size within the host) or fewer (because of resource competition or apparent competition *via* the immune system) is hard to predict; moreover, given that we know that the parasite shows strongly logistic growth within hosts, differences in initial population size might primarily influence when the parasite reaches its maximal abundance within the host, rather than the final density. While it is difficult to predict how developmental plasticity might impact spore yield, our results related to the proportion of spores that matured do not suggest developmental plasticity. Focusing on the warmer current temperature treatment (where there was a larger, but still non-significant, difference between infection levels for the 2 rearing treatments), spores from the cooler rearing treatment tended towards lower infection levels than those from the warmer rearing treatment (Fig. 1A). However, this lower infection level was associated with a lower proportion of spore maturation (Fig. 1C). We cannot come up with a plausible explanation for why an early infection environment with fewer individuals would reduce spore maturation, so we suspect this result was not driven by developmental plasticity. However, future investigations of transgenerational plasticity in parasites should be designed in a way that allows for disentangling transgenerational effects from developmental plasticity.

While parasite fitness was explained both by the effects of rearing and current temperatures, parasite virulence was exclusively determined by current temperatures. Specifically, parasites were more virulent (i.e. greater reduction in host fecundity and lifespan; Fig. 2) at higher temperatures, when the total number of spores was also higher (i.e. suggesting stronger within-host competition; Fig. 1B). This is in line with previous theoretical work suggesting that parasite virulence should be higher when intraspecific competition is intense (Lively, 2006). The absence of an effect of rearing temperatures suggests that parasite transgenerational plasticity had negligible influences on parasite virulence in this system. Instead, parasite virulence was mainly determined by the thermal ecology of host–parasite interactions in current environments, consistent with another recent study (Schampera *et al*., 2022).

Together, our results highlight the importance of incorporating parasite infection history and the potential for context-dependent transgenerational plasticity. Future research should consider how transgenerational plasticity contributes to parasite adaptation and its consequence on disease transmission, in order to better understand host–parasite interactions in changing environments.

## Data Availability

The data and code used for this study are available on GitHub (https://github.com/syuanjyunsun/parasite-transgen-exp).

## Supplementary material

For supplementary material accompanying this paper visit https://doi.org/10.1017/S0031182022001056. click here to view supplementary material

## References

[ref1] Altizer S, Dobson A, Hosseini P, Hudson P, Pascual M and Rohani P (2006) Seasonality and the dynamics of infectious diseases. Ecology Letters 9, 467–484. 16623732 10.1111/j.1461-0248.2005.00879.x

[ref2] Altizer S, Ostfeld RS, Johnson PTJ, Kutz S and Harvell CD (2013) Climate change and infectious diseases: from evidence to a predictive framework. Science 341, 514–519. 23908230 10.1126/science.1239401

[ref3] Bates D, Maechler M, Bolker B and Walker S (2015) Fitting linear mixed-effects models using lme4. *R package version* 1.0-6.

[ref4] Beits RA, Collins M, Hemming DL, Jones CD, Lowe JA and Sanderson MG (2011) When could global warming reach 4C? Philosophical Transactions of the Royal Society A: Mathematical. Physical and Engineering Sciences 369, 67–84. 10.1098/rsta.2010.0292 21115513

[ref5] Burgess SC and Marshall DJ (2014) Adaptive parental effects: the importance of estimating environmental predictability and offspring fitness appropriately. Oikos 123, 769–776.

[ref6] Cáceres CE, Tessier AJ, Duffy MA and Hall SR (2014) Disease in freshwater zooplankton: what have we learned and where are we going? Journal of Plankton Research 36, 326–333.

[ref7] Carlson CJ, Burgio KR, Dougherty ER, Phillips AJ, Bueno VM, Clements CF, Castaldo G, Dallas TA, Cizauskas CA, Cumming GS, Doña J, Harris NC, Jovani R, Mironov S, Muellerklein OC, Proctor HC and Getz WM (2017) Parasite biodiversity faces extinction and redistribution in a changing climate. Science Advances 3, e1602422. 28913417 10.1126/sciadv.1602422 PMC5587099

[ref8] Charmantier A, McCleery RH, Cole LR, Perrins C, Kruuk LEB and Sheldon BC (2008) Adaptive phenotypic plasticity in response to climate change in a wild bird population. Science (New York, N.Y.) 320, 800–803. 18467590 10.1126/science.1157174

[ref9] Clay PA, Dhir K, Rudolf VHW and Duffy MA (2019) Within-host priority effects systematically alter pathogen coexistence. The American Naturalist 193, 187–199. 10.1086/701126 30720357

[ref10] Decaestecker E, de Meester L and Ebert D (2002) In deep trouble: habitat selection constrained by multiple enemies in zooplankton. Proceedings of the National Academy of Sciences of the United States of America 99, 5481–5485. 11960005 10.1073/pnas.082543099 PMC122795

[ref11] Decaestecker E, Lefever C, de Meester L and Ebert D (2004) Haunted by the past: evidence for dormant stage banks of microparasites and epibionts of *Daphnia*. Limnology and Oceanography 49, 1355–1364.

[ref12] Duffy MA and Hunsberger KK (2019) Infectivity is influenced by parasite spore age and exposure to freezing: do shallow waters provide *Daphnia* a refuge from some parasites? Journal of Plankton Research 41, 12–16.

[ref13] Ebert D (1995) The ecological interactions between a microsporidian parasite and its host *Daphnia magna*. The Journal of Animal Ecology 64, 361.

[ref14] Ebert D (2005) Ecology, Epidemiology, and Evolution of Parasitism in Daphnia. Bethesda, MD: National Library of Medicine (USA), Center for Biotechnology Information.

[ref15] Galloway LF and Etterson JR (2007) Transgenerational plasticity is adaptive in the wild. Science 318, 1134–1136. 18006745 10.1126/science.1148766

[ref16] Gehman A-LM, Hall RJ and Byers JE (2018) Host and parasite thermal ecology jointly determine the effect of climate warming on epidemic dynamics. Proceedings of the National Academy of Sciences of the United States of America 115, 744–749. 29311324 10.1073/pnas.1705067115 PMC5789902

[ref17] Gervasi SS, Civitello DJ, Kilvitis HJ and Martin LB (2015) The context of host competence: a role for plasticity in host–parasite dynamics. Trends in Parasitology 31, 419. 26048486 10.1016/j.pt.2015.05.002 PMC4567474

[ref18] Harmon EA and Pfennig DW (2021) Evolutionary rescue *via* transgenerational plasticity: evidence and implications for conservation. Evolution & Development 23, 292–307. 33522673 10.1111/ede.12373

[ref19] Harvell CD, Mitchell CE, Ward JR, Altizer S, Dobson AP, Ostfeld RS and Samuel MD (2002) Climate warming and disease risks for terrestrial and marine biota. Science 296, 2158–2162. 12077394 10.1126/science.1063699

[ref20] Hoffmann AA and Sgrò CM (2011) Climate change and evolutionary adaptation. Nature 470, 479–485. 21350480 10.1038/nature09670

[ref21] Kunze C, Luijckx P, Jackson AL and Donohue I (2022) Alternate patterns of temperature variation bring about very different disease outcomes at different mean temperatures. eLife 11, e72861. doi: 10.7554/ELIFE.72861 35164901 PMC8846586

[ref22] Lenth RV (2021) Emmeans: estimated marginal means, aka least-squares means. R package version 1.7.1. R Foundation for Statistical Computing 34, 216–221.

[ref23] Little T, Birch J, Vale P and Tseng M (2007) Parasite transgenerational effects on infection. Evolutionary Ecology Research 9, 459–469.

[ref24] Lively CM (2006) The ecology of virulence. Ecology Letters 9, 1089–1095. 16972872 10.1111/j.1461-0248.2006.00969.x

[ref25] Manzi F, Agha R, Lu Y, Ben-Ami F and Wolinska J (2020) Temperature and host diet jointly influence the outcome of infection in a *Daphnia*–fungal parasite system. Freshwater Biology 65, 757–767.

[ref26] Martinez ME (2018) The calendar of epidemics: seasonal cycles of infectious diseases. PLoS Pathogens 14, e1007327. 30408114 10.1371/journal.ppat.1007327 PMC6224126

[ref27] Meng S, Tran TT, Delnat V and Stoks R (2021) Transgenerational exposure to warming reduces the sensitivity to a pesticide under warming. Environmental Pollution 284, 117217. 33915393 10.1016/j.envpol.2021.117217

[ref28] Michel J, Ebert D and Hall MD (2016) The trans-generational impact of population density signals on host–parasite interactions. BMC Evolutionary Biology 16, 1–12. 27887563 10.1186/s12862-016-0828-4 PMC5123254

[ref29] Mousseau TA and Fox CW (1998) The adaptive significance of maternal effects. Trends in Ecology & Evolution 13, 403–407. 21238360 10.1016/s0169-5347(98)01472-4

[ref30] Munday PL, Warner RR, Monro K, Pandolfi JM and Marshall DJ (2013) Predicting evolutionary responses to climate change in the sea. Ecology Letters 16, 1488–1500. 24119205 10.1111/ele.12185

[ref31] Nystrand M, Cassidy EJ and Dowling DK (2016) Transgenerational plasticity following a dual pathogen and stress challenge in fruit flies. BMC Evolutionary Biology 16, 1–11. 27567640 10.1186/s12862-016-0737-6 PMC5002108

[ref32] Paraskevopoulou S, Gattis S and Ben-Ami F (2022) Parasite resistance and parasite tolerance: insights into transgenerational immune priming in an invertebrate host. Biology Letters 18, 20220018. doi: 10.1098/RSBL.2022.0018 35382587 PMC8984330

[ref33] Pigeault R, Vézilier J, Nicot A, Gandon S and Rivero A (2015) Transgenerational effect of infection in *Plasmodium*-infected mosquitoes. Biology Letters 11, 20141025. doi: 10.1098/RSBL.2014.1025 25762571 PMC4387496

[ref34] Radchuk V, Reed T, Teplitsky C, van de Pol M, Charmantier A, Hassall C, Adamík P, Adriaensen F, Ahola MP, Arcese P, Miguel Avilés J, Balbontin J, Berg KS, Borras A, Burthe S, Clobert J, Dehnhard N, de Lope F, Dhondt AA, Dingemanse NJ, Doi H, Eeva T, Fickel J, Filella I, Fossøy F, Goodenough AE, Hall SJG, Hansson B, Harris M, Hasselquist D, Hickler T, Joshi J, Kharouba H, Martínez JG, Mihoub JB, Mills JA, Molina-Morales M, Moksnes A, Ozgul A, Parejo D, Pilard P, Poisbleau M, Rousset F, Rödel MO, Scott D, Senar JC, Stefanescu C, Stokke BG, Kusano T, Tarka M, Tarwater CE, Thonicke K, Thorley J, Wilting A, Tryjanowski P, Merilä J, Sheldon BC, Pape Møller A, Matthysen E, Janzen F, Dobson FS, Visser ME, Beissinger SR, Courtiol A and Kramer-Schadt S (2019) Adaptive responses of animals to climate change are most likely insufficient. Nature Communications 10, 1–14. 10.1038/s41467-019-10924-4 PMC6650445 31337752

[ref35] R Development Core Team (2014) R: A Language and Environment for Statistical Computing. Vienna, Austria: R Foundation for Statistical Computing.

[ref36] Röder G, Rahier M and Naisbit RE (2008) Counter-intuitive developmental plasticity induced by host quality. Proceedings of the Royal Society B: Biological Sciences 275, 879–885. 10.1098/rspb.2007.1649 PMC2599943 18198142

[ref37] Roth O and Landis SH (2017) Trans-generational plasticity in response to immune challenge is constrained by heat stress. Evolutionary Applications 10, 514–528. 28515783 10.1111/eva.12473 PMC5427669

[ref38] Schampera C, Agha R, Manzi F and Wolinska J (2022) Parasites do not adapt to elevated temperature, as evidenced from experimental evolution of a phytoplankton–fungus system. Biology Letters 18, 20210560. doi: 10.1098/RSBL.2021.0560 35168375 PMC8847893

[ref39] Searle CL, Ochs JH, Cáceres CE, Chiang SL, Gerardo NM, Hall SR and Duffy MA (2015) Plasticity, not genetic variation, drives infection success of a fungal parasite. Parasitology 142, 839–848. 25711627 10.1017/S0031182015000013

[ref40] Shama LNS, Strobel A, Mark FC and Wegner KM (2014) Transgenerational plasticity in marine sticklebacks: maternal effects mediate impacts of a warming ocean. Functional Ecology 28, 1482–1493.

[ref41] Shocket MS, Vergara D, Sickbert AJ, Walsman JM, Strauss AT, Hite JL, Duffy MA, Cáceres CE and Hall SR (2018) Parasite rearing and infection temperatures jointly influence disease transmission and shape seasonality of epidemics. Ecology 99, 1975–1987. 29920661 10.1002/ecy.2430

[ref42] Stewart Merrill TE, Hall SR, Merrill L and Cáceres CE (2019) Variation in immune defense shapes disease outcomes in laboratory and wild *Daphnia*. Integrative and Comparative Biology 59, 1203–1219. 31141120 10.1093/icb/icz079

[ref43] Sun S-J, Catherall AM, Pascoal S, Jarrett BJM, Miller SE, Sheehan MJ and Kilner RM (2020) Rapid local adaptation linked with phenotypic plasticity. Evolution Letters 4, 345–359. 32774883 10.1002/evl3.176 PMC7403679

[ref44] Sun S-J, Dziuba MK, Jaye RN and Duffy MA (2022) Temperature modifies trait-mediated infection outcomes in a *Daphnia*–fungal parasite system. bioRxiv. doi: 10.1101/2022.06.03.494706. PMC9900708 36744571

[ref45] Therneau T (2012) Coxme: mixed effects Cox models. *R package version*, 2.2-3.

[ref46] Tran TT, Janssens L, Dinh KV and Stoks R (2019) An adaptive transgenerational effect of warming but not of pesticide exposure determines how a pesticide and warming interact for antipredator behaviour. Environmental Pollution 245, 307–315. 30447473 10.1016/j.envpol.2018.11.022

[ref47] Tseng M (2006) Interactions between the parasite's previous and current environment mediate the outcome of parasite infection. The American Naturalist 168, 565–571. 10.1086/507997 17004228

[ref48] Vale PF, Stjernman M and Little TJ (2008) Temperature-dependent costs of parasitism and maintenance of polymorphism under genotype-by-environment interactions. Journal of Evolutionary Biology 21, 1418–1427. 18557795 10.1111/j.1420-9101.2008.01555.x

[ref49] Wong BBM and Candolin U (2015) Behavioral responses to changing environments. Behavioral Ecology 26, 665–673.

