# Supplementary material for: Transgenerational plasticity alters parasite fitness in changing environments
Source: Parasitology. 2022 Aug 4;149(11):1515–20. doi: 10.1017/S0031182022001056 (PMC10090760; doi:10.1017/S0031182022001056)
Supplement: Supplementary file 1 [file S0031182022001056sup001.docx]

Supplementary materials:

Figure S1.

An example of mature and immature spores of the parasite *Metschnikowia bicuspidata*. At maturity, the needle-shaped ascospores can easily be distinguished from immature spores, which are still conidia or needle-like spores lacking a sharp edge and dark band in the center (see also Supplementary Data in Stewart Merrill et al. 2019). Photo credit: Marcin K. Dziuba.
